# Association of heart rate recovery with mortality risk in a 72-month survival analysis

**DOI:** 10.1590/0102-311XEN113025

**Published:** 2026-05-01

**Authors:** Bruno Margueritte Costa, Cleverson Motin, João Vicente Marinho de Sousa, Nilo Massaru Okuno, Adalberto Ferreira-Junior

**Affiliations:** 1 Universidade Estadual de Ponta Grossa, Ponta Grossa, Brasil.

**Keywords:** Autonomic Nervous System Diseases, Exercise Test, Cardiovascular Diseases, Doenças do Sistema Nervoso Autônomo, Teste de Esforço, Doenças Cardiovasculares, Enfermedades del Sistema Nervioso Autónomo, Prueba de Esfuerzo, Enfermedades Cardiovasculares

## Abstract

This study aimed to investigate whether heart rate recovery (HRR) is a predictor of all-cause mortality in a 72-month survival analysis. In this cohort study, 578 individuals who underwent a maximal exercise test at a university hospital between August 2012 and August 2018 were followed for up to 72 months. Participants performed a maximal incremental treadmill test, followed by an active recovery period phase to evaluate their HRR. When the decay of heart rate totaled ≥ 13bpm in the first minute post-exercise a normal HRR was considered, whereas an abnormal HRR was defined as ≤ 12bpm. Of the 578 individuals, 371 (64.2%) had normal HRR, whereas 207 (35.8%) had an abnormal HRR. The survival analysis showed significantly higher 72-month survival in the normal HRR group (93.8%) than in the abnormal HRR group (81.6%), with a 3.16 hazard ratio (95%CI: 1.86-5.35). Individuals with abnormal HRR were generally older, had a greater burden of comorbidities, shorter time to exhaustion, lower peak heart rate, and higher heart rate 60 seconds after the exercise. Thus, HRR predicted 72-month all-cause mortality. These findings suggest the use of HRR as a simple, non-invasive prognostic tool in clinical practice, particularly for stratifying mortality risk.

## Abstract (pt)

O estudo teve como objetivo investigar se a frequência cardíaca de recuperação (FCR) é um preditor de mortalidade por todas as causas ao longo de 72 meses. Neste estudo de coorte, 578 indivíduos submetidos a um teste de esforço máximo em um hospital universitário entre agosto de 2012 e agosto de 2018 foram acompanhados por até 72 meses. Os participantes realizaram um teste incremental máximo em esteira, seguido por uma fase de recuperação ativa para avaliar a FCR. A FCR foi considerada normal quando o declínio da frequência cardíaca foi ≥ 13bpm no primeiro minuto pós-exercício, enquanto a FCR anormal foi definida como ≤ 12bpm. Entre os 578 indivíduos, 371 (64,2%) apresentaram FCR normal e 207 (35,8%) apresentaram FCR anormal. A análise de sobrevida demonstrou sobrevida após 72 meses significativamente maior no grupo com FCR normal (93,8%) em comparação ao grupo com FCR anormal (81,6%), com uma razão de risco de 3,16 (IC95%: 1,86-5,35). De modo geral, indivíduos com FCR anormal eram mais velhos, apresentavam maior carga de comorbidades, menor tempo até a exaustão, menor frequência cardíaca de pico e maior frequência cardíaca aos 60 segundos pós-exercício. Em conclusão, a FCR foi um preditor de mortalidade por todas as causas ao longo de 72 meses. Esses achados sugerem o uso da FCR como uma ferramenta prognóstica simples e não invasiva na prática clínica, particularmente para a estratificação do risco de mortalidade.

## Abstract (es)

Este estudio se propuso investigar si la recuperación de la frecuencia cardíaca (RFC) es un indicador de la mortalidad por todo tipo de causas en un plazo de 72 meses. En este estudio de cohorte, se hizo un seguimiento de 72 meses a 578 personas que se sometieron a una prueba de esfuerzo máxima en un hospital universitario entre agosto de 2012 y agosto de 2018. Los participantes realizaron una prueba incremental máxima en cinta rodante, seguida de una fase de recuperación activa para evaluar la RFC. Se consideró que la RFC era normal cuando la disminución de la frecuencia cardíaca era ≥ 13lpm en el primer minuto después del ejercicio, mientras que se definió como anómala cuando era ≤ 12lpm. De las 578 personas, 371 (64,2%) tenían una RFC normal, mientras que 207 (35,8%) tenían una RFC anormal. El análisis de supervivencia demostró una supervivencia significativamente mayor a los 72 meses en el grupo con RFC normal (93,8%) en comparación con el grupo con RFC anormal (81,6%), con una razón de riesgo de 3,16 (IC95%: 1,86-5,35). Las personas con RFC anormal eran generalmente mayores, tenían una mayor carga de comorbilidades, un tiempo más corto hasta el agotamiento, una frecuencia cardíaca máxima más baja y una frecuencia cardíaca más alta a los 60 segundos después del ejercicio. En conclusión, se observó que la RFC era un predictor de mortalidad por todas las causas en un plazo de 72 meses. Estos hallazgos sugieren el uso de la RFC como una herramienta pronóstica sencilla y no invasiva en la práctica clínica, especialmente para estratificar el riesgo de mortalidad.

## Introduction

Heart rate recovery (HRR) is a non-invasive tool to evaluate cardiac autonomic control after exercise ^1^. This method has become widely used to investigate active or passive recovery. During physical exertion, heart rate increases due to vagal withdrawal and enhanced sympathetic activation. Conversely, after exercise cessation, heart rate declines via rapid vagal reactivation and gradual sympathetic activity withdrawal ^2^. A slower post-exercise heart rate decay indicates impaired autonomic control and has been associated with adverse cardiovascular outcomes ^3^.

Autonomic dysfunction may be associated with an increased cardiovascular risk. A previous study showed that a decline of ≤ 12bpm in the first minute of active recovery was considered an abnormal HRR and associated with mortality ^1^. Similar associations have been reported in healthy populations ^4^, including an elevated risk of sudden death ^5^.

Considering its simplicity, cost-effectiveness, and clinical utility, HRR has become an important prognostic tool in exercise testing. However, to our knowledge, no studies have investigated whether HRR can predict mortality in the Brazilian population. Brazil has a public health system that needs validated prognostic indicators to identify risks, improve clinical care, and reduce costs; thus, evaluating HRR may configure an interesting tool due to its low cost and applicability. Therefore, this study aimed to investigate whether HRR predicts 72-month all-cause mortality in a Brazilian high-risk cohort. We hypothesized that abnormal HRR is associated with a higher risk of mortality due to autonomic dysfunction.

## Methods

### Individuals

Individuals who had been referred for cardiology evaluation due to their high-risk of a cardiovascular event were included in this cohort study. They performed a maximal exercise test at the University Hospital of the State University of Ponta Grossa (HU/UEPG, acronym in Portuguese) between August 2012 and August 2018. Initially, 646 participants were assessed. Of these, 34 individuals were excluded from the final analysis due to incomplete data; another 34 individuals were excluded for being aged under 35 years, resulting in a final sample of 578 participants. All participants signed a informed consent form agreeing to participate in this study. This study was approved by the Ethics Committee for Research on Human Subjects of the State University of Ponta Grossa (protocol 3282924) and conducted in accordance with the latest edition of the *Declaration of Helsinki*.

### Exercise test protocol

Participants performed a maximal exercise test on a treadmill (Centurion 200, Micromed; https://micromed.health/) under the Bruce ^6^ or ramp protocols ^7^. The Bruce protocol is characterized by an increment in velocity or slope every three minutes. In turn, the ramp protocol is characterized by small and constant increments in velocity and slope according to individuals’ age and sex. Participants were encouraged to reach their maximum capacity. After exhaustion, they performed a period of active recovery in which the treadmill was maintained at 2.4km/h and 2.5% slope. Throughout the test and recovery, participants’ heart rate was continuously monitored by an electrocardiograph (ErgoPC Elite, Micromed). The routine medications that influenced heart rate were discontinued before the test following Brazilian guidelines ^8^.

### Outcome

The individuals were followed for 72 months. All-cause mortality was chosen as the main outcome in this study. It was analyzed in individuals with normal and abnormal HRR. HRR was defined as the difference between peak heart rate and heart rate at 60 seconds into active recovery. A normal HRR was considered if the HRR at the first post-exercise minute was ≥ 13bpm, whereas an abnormal HRR was considered if HRR was ≤ 12bpm ^1^. Mortality was identified using medical records, and phone contact confirmed survival. All-cause mortality according to the number of risk factors was chosen as the secondary outcome in this study. The following risk factors were considered in this research: abnormal HRR, diabetes, hypertension, obesity, smoking, dyslipidemia, and older age.

### Statistical analysis

Data distribution was assessed by the Kolmogorov-Smirnov test. Continuous variables are shown as means and standard deviations. The groups were compared using the independent t- or Mann-Whitney tests depending on normality. Categorical variables are shown as absolute and relative frequencies. Between-group comparisons were performed using the chi-squared test. A Kaplan-Meier curve was used to verify the survival proportion during follow-up. Hazard ratios (HR) and 95% confidence intervals (95%CI) were also calculated. All statistical procedures were performed on GraphPad Prism, version 8.0.1 (https://www.graphpad.com/), with statistical significance set at p < 0.05.

## Results

A total of 578 individuals completed the test and were followed for 72 months. Among them, 371 (64.2%) had a normal HRR, whereas 207 (35.8%) had an abnormal HRR. Table 1 shows these individuals’ clinical characteristics. Those with an abnormal HRR were older (p < 0.001) and had a higher prevalence of females (p = 0.04), diabetes mellitus (p < 0.001), systemic arterial hypertension (p < 0.001), and obesity (p = 0.01).

**Table 1:** Participants’ characteristics.

Characteristics	Normal HRR (n = 371) n (%)	Abnormal HRR (n = 207) n (%)	p-value
Age (years) [mean ± SD]	56.2 ± 10.0	62.4 ± 9.2	< 0.001 *
Female gender	209 (56.3)	135 (65.2)	0.04 *
Body mass (kg) [mean ± SD]	76.6 ± 16.6	79.2 ± 18.7	0.16
Previous MI	19 (5.1)	6 (2.9)	0.22
Previous CR	29 (7.8)	13 (6.3)	0.50
Diabetes	72 (19.4)	79 (38.2)	< 0.001 *
Hypertension	222 (59.8)	168 (81.2)	< 0.001 *
Smoking	74 (20.0)	37 (17.9)	0.54
Dyslipidemia	43 (11.6)	29 (14.0)	0.40
Obesity	121 (32.6)	87 (42.0)	0.01 *

CR: coronary revascularization; HRR: heart rate recovery; MI: myocardial infarction; SD: standard deviation.

* p < 0.05.

Table 2 describes the analyzed variables in the maximal exercise test and recovery period. Individuals with an abnormal HRR had shorter time to exhaustion (p < 0.001), lower peak heart rate (p < 0.001), higher heart rate at 60 seconds after exercise (p < 0.001), and lower HRR (p < 0.001) than those with a normal HRR.

**Table 2:** Performance in the maximal exercise test and recovery period.

	Normal HRR (n = 371) Mean ± SD	Abnormal HRR (n = 207) Mean ± SD	p-value
Test duration (s)	417.3 ± 119.8	315.6 ± 117.0	< 0.001 *
Heart rate peak (bpm)	147.5 ± 13.8	140.6 ± 16.6	< 0.001 *
Heart rate 60s (bpm)	124.5 ± 14.4	133.8 ± 15.8	< 0.001 *
HRR (bpm)	23.0 ± 8.5	6.8 ± 5.0	< 0.001 *

HRR: heart rate recovery; SD: standard deviation.

* p < 0.05.

**Figure 1:**
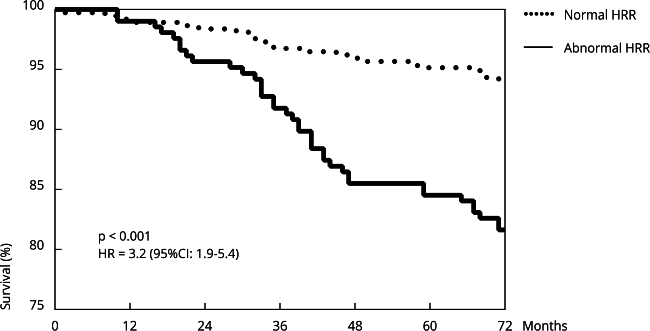
Kaplan-Meier curve showing the difference in survival between individuals with normal and abnormal heart rate recovery (HRR) during the follow-up period.

Follow-up found 61 deaths, of which 38 (62.3%) occurred in individuals with an abnormal HRR. The Kaplan-Meier curve shows the survival rate in individuals with normal and abnormal HRRs throughout follow-up (Figure 1). Individuals with a normal HRR had a higher survival rate (93.8%) than those with an abnormal HRR (81.6%) (p < 0.001), with a HR of 3.2 (95%CI: 1.9-5.4).

**Figure 2:**
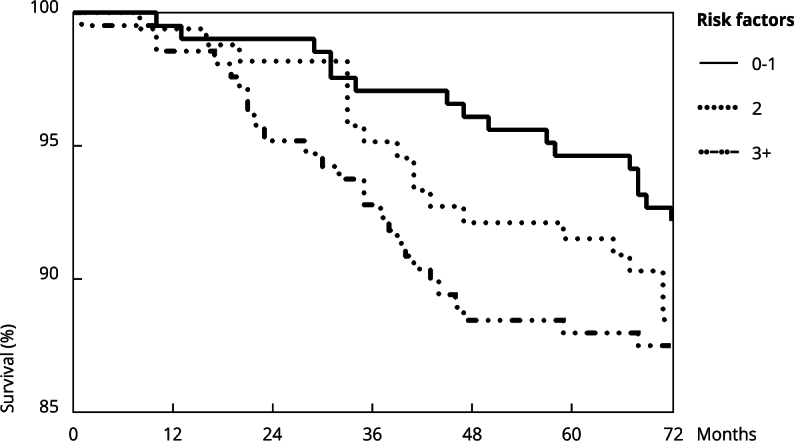
Kaplan-Meier curve showing the survival rate according to the number of risk factors (abnormal heart rate recovery, diabetes, hypertension, obesity, smoking, dyslipidemia, and older age).

This study found no significant differences in survival rate according to the number of risk factors during follow-up (p = 0.24) (Figure 2).

95%CI: 95% confidence interval; HR: hazard ratio.

## Discussion

This study aimed to investigate HRR as a predictor of mortality within a 72-month period. It observed that individuals with an abnormal HRR showed significantly lower survival rates (81.6%) than those with normal HRR (93.8%). Additionally, individuals with an abnormal HRR were older and had a higher prevalence of comorbidities and shorter time to exhaustion. These findings suggest that HRR is a relevant prognostic indicator for mortality risk in the Brazilian population, reinforcing the importance of this evaluation in clinical practice.

Our findings are in line with previous studies on HRR over similar follow-up lengths. Cole et al. ^1^ and Vivekananthan et al. ^3^ reported comparable mortality rates in participants with abnormal HRRs (18.8% and 19.3%, respectively - and HR of 4.0 [95%CI: 3.0-5.2] and 2.5 [95%CI: 2.0-3.1]), whereas our findings showed the death of 18.4% of patients with abnormal HRRs (HR = 3.2; 95%CI: 1.9-5.4). such consistency across studies reinforces the reliability of HRR as a prognostic indicator for 72-month all-cause mortality.

Patients with abnormal HRRs shared some characteristics, such as older age, diabetes, hypertension, and obesity. Some studies have observed that these characteristics are associated with cardiac autonomic dysfunction ^9^ ^,^ ^10^ ^,^ ^11^ ^,^ ^12^ and abnormal HRRs ^1^ ^,^ ^3^. Thus, people having both abnormal HRRs and comorbidities should be regarded as high-risk individuals and may benefit from target monitoring and intervention in clinical practice.

However, combining risk factors obtained no difference in the 72-month follow-up. Clinical evaluations can easily measure traditional risk factors such as diabetes and hypertension. Medical care could improve such circumstances by medications and behavior change ^13^. In turn, HRR remains underrated in clinical scenario, although autonomic dysfunction constitutes a relevant health concern. Although combining risk factors offers an interesting way to investigate long-term survival rates, HRR seemed to constitute a better prognostic value in the 72-month follow-up in this study.

HRR is a non-invasive, inexpensive, and accessible tool, making it highly feasible for routine use in various healthcare settings, including the Brazilian Unified National Health System. Its application may enhance risk stratification, improve preventive care, and help to optimize resource allocation.

This study has some limitations. The analysis only considered all-cause mortality, not including specific causes of death. Additionally, its single-center design may limit its generalizability. However, as the chosen hospital serves multiple municipalities, this limitation is somewhat mitigated. Finally, the participants’ physical activity level was not evaluated.

HRR was confirmed a predictor of mortality within 72 months. These findings support the use of HRR as a valuable clinical marker for identifying high-risk individuals. Its low cost, non-invasiveness, and ease of implementation make it a compelling tool for routine clinical practice, especially within public healthcare systems.

## Data Availability

The research data are available upon request to the corresponding author.
